# Retinal structure and vessel density changes in cerebral small vessel disease

**DOI:** 10.3389/fnins.2024.1288380

**Published:** 2024-02-26

**Authors:** Dandan Wang, Lina Wang, Jinjin Wang, Yang Du, Kaiyue Wang, Meizi Wang, Liu Yang, Xingquan Zhao

**Affiliations:** ^1^Department of Neurology, Beijing Tiantan Hospital, Capital Medical University, Beijing, China; ^2^Department of Ophthalmology, Beijing Tiantan Hospital, Capital Medical University, Beijing, China; ^3^Department of Neurology, Peking University Sixth Hospital, Peking University Institute of Mental Health, Beijing, China; ^4^China National Clinical Research Center for Neurological Diseases, Beijing, China

**Keywords:** cerebral small vessel disease (CSVD), retinal structure, retinal vessel density, diagnosis, neuro-ophtalmology

## Abstract

**Background:**

Cerebral small vessel disease (CSVD) attaches people’s attention in recent years. In this study, we aim to explore retinal structure and vessel density changes in CSVD patients.

**Methods:**

We collected information on retinal metrics assessed by optical coherence tomography (OCT) and OCT angiography and CSVD characters. Logistic and liner regression was used to analyze the relationship between retinal metrics and CSVD.

**Results:**

Vessel density of superficial retinal capillary plexus (SRCP), foveal density- 300 length (FD-300), radial peripapillary capillary (RPC) and thickness of retina were significantly lower in CSVD patients, the difference only existed in the thickness of retina after adjusted relevant risk factors (OR (95% CI): 0.954 (0.912, 0.997), *p* = 0.037). SRCP vessel density showed a significant downward trend with the increase of CSVD scores (β: −0.087, 95%CI: −0.166, −0.008, *p* = 0.031). SRCP and FD-300 were significantly lower in patients with lacunar infarctions and white matter hypertensions separately [OR (95% CI): 0.857 (0.736, 0.998), *p* = 0.047 and OR (95% CI): 0.636 (0.434, 0.932), *p* = 0.020, separately].

**Conclusion:**

SRCP, FD-300 and thickness of retina were associated with the occurrence and severity of total CSVD scores and its different radiological manifestations. Exploring CSVD by observing alterations in retinal metrics has become an optional research direction in future.

## Background

Cerebral small vessel disease (CSVD) has increasingly attracted people’s attention in recent years. In China, lacunar infarction (LI) caused by CSVD accounts for 25–50% of ischemic strokes (Chinese Medical Association of Neurology, 2022). Lam and colleagues found the incidence of CSVD is higher in low- and middle-income countries, and the proportion of moderate-to-severe white matter lesions increases progressively in community individuals, stroke patients, and dementia patients (Lam et al., 2023). Generally, most CSVD neuroimaging abnormalities have nonspecific manifestations. The clinical features of CSVD vary from non-symptom to stroke, dementia, depression, and gait disorders, and some of the CSVD patients leave a poor prognosis (The LADIS Study Group et al., 2011; Zeestraten et al., 2017; Zanon Zotin et al., 2021). So how to screen and treat CSVD early and reduce the poor outcome seems an urgent issue to doctors.

Retinal arteries and intracranial small arteries share a common origin in the internal carotid artery, with similar embryonic origins and anatomical features. The blood-retinal barrier is structurally and functionally similar to the blood–brain barrier as well. Previous studies have demonstrated the close relationship between retinal microvascular abnormality and stroke (Hughes et al., 2016; Umemura et al., 2017; Zhang et al., 2021). As a result, retinal vascular changes can reflect intracranial small vessel changes to some extent. Therefore, this study intends to explore retinal structure and vessel density changes in CSVD patients and the correlation between these changes and different CSVD radiological manifestations and clinical presentations. This will provide a new direction and approach for the screen and diagnosis of CSVD.

## Methods

### Study population

The study enrolled patients with cerebrovascular diseases as their tentative diagnosis hospitalized in Beijing Tiantan Hospital from March 2021 to August 2022. The study has been approved by the Ethics Committee of Beijing Tiantan Hospital (KY2021-064-02), and all enrolled patients have signed informed consent forms. The specific inclusion and exclusion criteria are as follows:

Inclusion criteria: (1) Age over 18 years old; (2) Patient diagnosis of CSVD or control by their neuroimaging features and clinical features; (3) Patient can cooperate with a complete medical history inquiry, laboratory tests, ophthalmological examinations, and head magnetic resonance imaging (MRI) and other related examinations; (4) mRS (modified Rankin Scale) score of 1 or lower before admission; and (5) Patient or family member is informed and cooperate with the examinations.

Exclusion criteria: (1) Age less than or equal to 18 years old; (2) History of hereditary CSVD; (3) History of severe eye diseases such as high myopia, glaucoma, or macular degeneration; (4) History of diabetes and a random blood glucose level higher than 11.1 mmoL/L at the first measurement during admission; (5) Patient undergoing surgical treatment for confirmed vascular stenosis during this admission; (6) Presence of metal or other foreign bodies in the body or other reasons that prevent the completion of head MRI; (7) Patient with hemiplegia, sensory aphasia, consciousness disorders, or other conditions that make them unable to cooperate with ophthalmological examinations, or patients with unclear imaging results that cannot be interpreted; (8) Patient with severe complicated diseases of the heart, liver, kidney, hematopoietic system, or tumors, with an expected life expectancy of less than 1 year; and (9) Patient or family members who refuse to participate in the study.

### Baseline data and medical history collection

On the day of enrollment, the physician collected general baseline information and epidemiological data from the participants, such as gender, age, smoking history, alcohol consumption history, medical history of cerebrovascular disease, hypertension, diabetes mellitus, hyperlipidemia and cognitive impairment. Basic physical examinations were completed at the same time, including height, weight, blood pressure measurement, and neurological examinations. Relevant personal history and disease definitions are as follows:

Smoking history: Currently smoking or quit smoking less than 5 years ago.
Alcohol consumption history: Currently drinking or quit drinking less than 5 years ago.
Body Mass Index (BMI): Weight (kg) / height (m) squared.
Cerebrovascular history: A history of ischemic stroke, hemorrhagic stroke or transit ischemic attack.
Hypertension history: Without medication, blood pressure levels measured 3 times on different days, systolic blood pressure (SBP) ≥140 mmHg or (and) diastolic blood pressure (DBP) ≥90 mmHg is considered hypertension. This includes patients with a previous diagnosis of hypertension or those currently taking antihypertensive drugs.
Diabetes mellitus history: A history of diabetes, or currently receiving hypoglycemic medication or insulin therapy, or a previous fasting blood glucose level of ≥7.0 mmol/L and/or a 2-h postprandial blood glucose level of ≥11.1 mmol/L.
Hyperlipidemia history: A history of hyperlipidemia, or currently receiving lipid-lowering drug therapy, or a previous serum low-density lipoprotein cholesterol (LDL-C) level of ≥3.37 mmol/L, or high-density lipoprotein cholesterol (HDL-C) level of <1.04 mmol/L, or triglyceride (TG) level of ≥1.7 mmol/L, or total cholesterol (TC) level of ≥5.17 mmol/L.
Cognitive impairment history: A history of cognitive impairment, which including mild cognitive impairment, dementia such as Alzheimer’s Disease, Lewy bodies-related dementia, frontotemporal dementia, and vascular dementia. All diagnoses of cognitive impairment must be acknowledged by neurologists or internists and conform to internationally recognized diagnostic criteria (Albert et al., 2011; McKhann et al., 2011).

### Laboratory tests

For all enrolled patients, venous blood from the antecubital vein was collected in the early morning on an empty stomach the day after admission. Blood routine, blood biochemistry, and glycated hemoglobin were measured, mainly recording the patient’s fasting blood glucose (FBG) and LDL-C. Fingerstick blood glucose measurements were taken postprandially and randomly if diabetes is suspected and recorded.

### Neuroimaging examination

Head MRI scans were performed using the American SIGNA HDe machine (slice thickness 5.00 mm/6.00 mm, FOV diameter 240 mm, scanning matrix 256 × 256) or the German SIEMENS Verio machine (slice thickness 5.00 mm/6.00 mm, FOV diameter 240 mm, scanning matrix 156 × 156). Sequences completed include T1, T2, DWI, ADC, FLAIR, SWI, and MRA. Based on the imaging results, two neuroimaging and neurology physicians read the images back-to-back, and recorded the scores for each CSVD imaging lesion (Table 1) (Staals et al., 2014; Goldstein et al., 2019). If the scores are inconsistent, a third professional neuroimaging physician performs a third independent interpretation and determines the final score.

**Table 1:** Total CSVD scores.

Imaging features	Score
LI	1
CMB	1
Moderate to severe PVS	1
Moderate to severe subcortical/periventricular WMH	1
Total:	4

LI, lacunar infarcts; CMB, cerebral microbleeds; PVS, perivascular spaces; WMH, white matter hyperintensities.

### Ophthalmological examinations

Enrolled patients underwent optical coherence tomography (OCT) and optical coherence tomography angiography (OCTA) examinations during hospitalization. The Avanti RTVue XR OCT System (Optovue, Inc., Fremont, CA, United States; Version: 2017.1.0.155) was used with patients seated in a relaxed position. Retinal thickness (RT), retinal average ganglion cell complex (GCC), retinal nerve fiber layer (RNFL) thickness was scanned by the center of macular fovea (diameter, 6 mm) and optic papilla (diameter, 3.45 mm) separately. The radial peripapillary capillary (RPC) scan was centered on the optic papilla (diameter, 4.5 mm) with the scanning depth spanning from the internal limiting membrane to the layer of nerve fiber. The superficial retinal capillary plexus (SRCP) was centered on the macular fovea (diameter, 3 mm; depth of the internal limiting membrane to 10 μm above the inner plexiform layers). The deep retinal capillary plexus (DRCP) was centered on the macular fovea (diameter, 3 mm), with a depth of 10 μm above the inner plexiform layers to within 10 μm below the outer plexiform layers. Vessel density was determined as the percentage of the local area occupied by blood vessels. RPC density refers to the density of blood vessels inside the optic disc area containing only capillaries. FD-300 vessel density is defined as foveal vessel density in a 300-μm wide zone around the Foveal Avascular Zone (FAZ) (Campbell et al., 2017; Inanc et al., 2019; Ma et al., 2022; Tang et al., 2022). The ophthalmic results of any one eye of the patient (first data from the left eye, right eye was used if left eye is unavailable) were used for later statistical analysis.

### Other examinations

If the enrolled patient agrees and cooperates, a brief mental state examination (MMSE) could be performed during the hospitalization to preliminarily evaluate cognitive function.

### Statistical analysis

The statistical analyses were performed using SPSS 24.0 software. Normally distributed measurement data are expressed as means± standard deviation, and independent sample *t*-tests are used for inter-group comparisons. Skewed distribution measurement data are expressed as median (percentage 25%- percentage 75%), and Wilcoxon tests are used for inter-group comparisons. Count data are expressed as rates (%), and chi-square tests were used for inter-group comparisons. Regression analysis is used to evaluate the effects of different ophthalmological results on different CSVD imaging manifestations and scores, calculating OR values and 95% CI for binary logistic regression and beta and 95% CI for liner regression, with *p* < 0.05 considered statistically significant.

## Results

**Figure 1:**
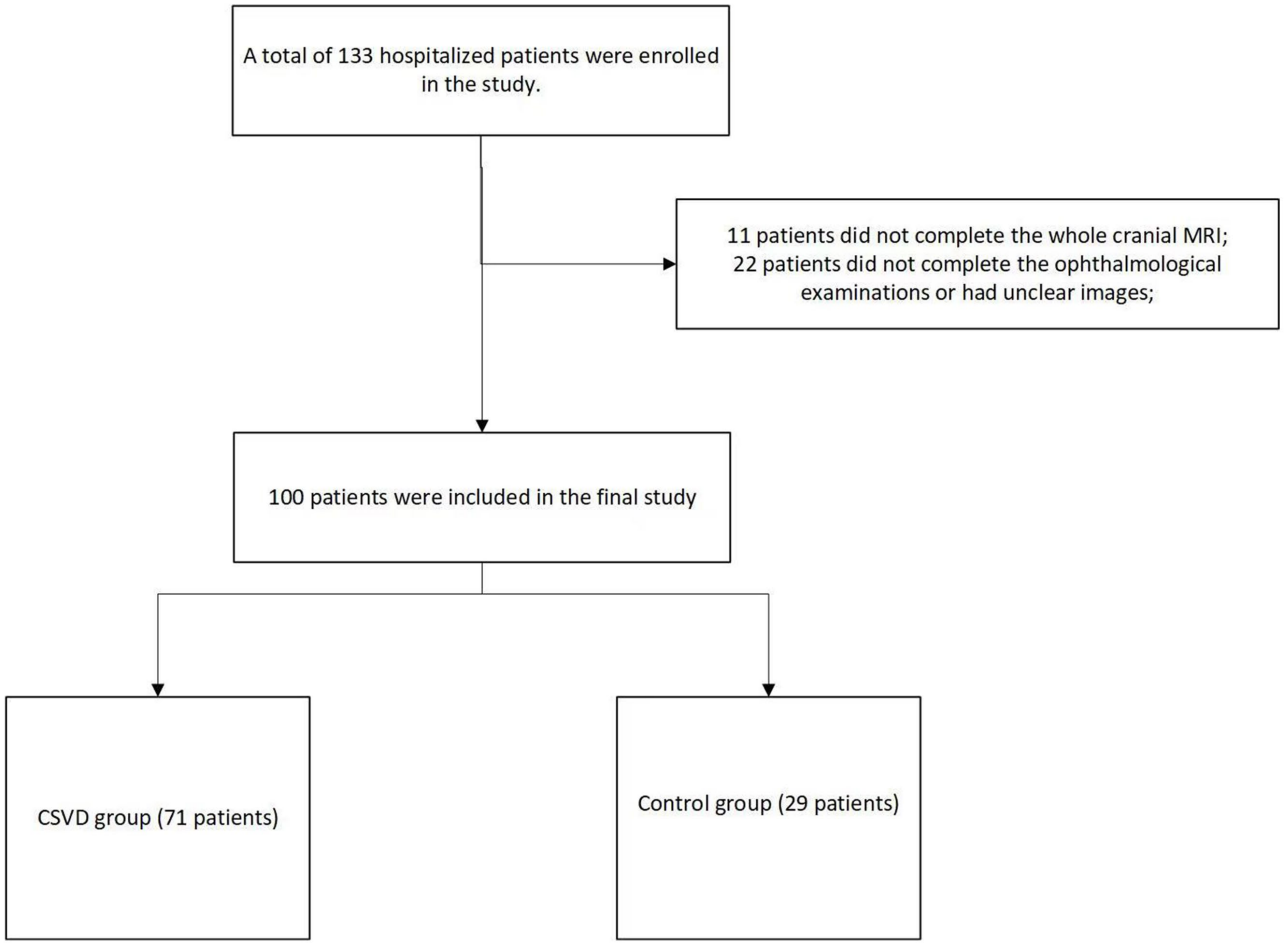
The flowchart of the study.

A total of 133 hospitalized patients were enrolled in the study. Among them, 11 patients did not complete the new cranial MRI examination during their hospitalization, and 22 patients did not complete the ophthalmological examinations or had unclear images. Finally, 100 patients were included in the final study. In our study population, there were 84 males and 16 females, with an average age of 53.6 ± 12.8 years. Based on their total CSVD scores, they were divided into a group with no CSVD imaging manifestations, recorded as the control group (29 patients), and a group with CSVD imaging manifestations, recorded as the CSVD group (71 patients) (Figure 1).

Comparing the basic epidemiological data and laboratory examinations of the two groups of patients, we found that the patients in the CSVD group were older, had a higher proportion of medical history of hypertension, and had higher admission systolic blood pressure than the control group, with statistical differences. There were no statistical differences between the two groups in terms of gender, smoking history, alcohol consumption history, BMI, medical history of diabetes mellitus、hyperlipidemia、 cognitive impairment, post-admission diastolic blood pressure, fast blood glucose, glycated hemoglobin, and LDL-C levels. Due to the small number of patients participating in cognitive function evaluation, there were no differences in MMSE and MoCA scores between the two groups (Table 2).

**Table 2:** Basic characteristics of patients between groups.

	Missing (*n*)	CSVD group	Control group	*p* value
Number		71	29	
Age (quartile), y		55.0 (50.0, 64.0)	54.0 (37.0, 58.5)	0.046
Male (*n*), %	0	62 (87.3)	22 (75.9)	0.156
Smoking (*n*), %	1	39 (55.7)	13 (44.8)	0.324
Alcohol consumption (*n*), %	1	35 (50.0)	11 (37.9)	0.273
BMI (x ± s)		25.4 ± 3.2	25.3 ± 3.5	0.884
Medical history of (*n*), %				
CVD	1	9 (12.9)	3 (10.3)	0.727
HBP	1	49 (70.0)	11 (37.9)	0.003
DM	1	7 (10.0)	3 (10.3)	0.959
Hyperlipidemia	1	21 (30.0)	10 (34.5)	0.662
CI	1	2 (2.9)	0 (0.0)	0.358
SBP (x ± s), mmHg		148.2 ± 20.7	136.6 ± 17.9	0.010
DBP (x ± s), mmHg		87.1 ± 12.1	90.5 ± 14.5	0.274
GHb (quartile), %		5.8 (5.5, 6.1)	5.6 (5.4, 6.2)	0.380
FBG (quartile), mmol/l		4.7 (4.3, 5.1)	4.6 (4.2, 5.1)	0.356
LDL-C (quartile), mmol/l		2.0 (1.6, 2.6)	2.4 (1.5, 2.8)	0.646
MMSE (quartile)	77	27.0(22.0,28.2)	26.0(24.5,29.0)	0.691
MoCA (quartile)	78	21.6 ± 5.2	24.0 ± 3.7	0.399

BMI, body mass index; CVD, cerebrovascular disease; CI, cognitive impairment; SBP, systolic blood pressure; DBP, diastolic blood pressure; GHb, glycated hemoglobin; FBG, fasting blood glucose; LDL-C, low-density lipoprotein cholesterol.

By analyzing and comparing the various ophthalmological indicators of the two groups of patients, we found that the vessel density of SRCP (Odd Ratio (OR) (95% Confidential Interval (CI)): 0.831 (0.704, 0.982), *p* = 0.030), FD-300 (OR (95% CI): 0.668 (0.460, 0.970), *p* = 0.034), and RPC (OR (95% CI): 0.910 (0.832, 0.996), *p* = 0.040) in the CSVD group were lower than those in the control group. However, after adjusting for gender, age, and medical histories, the statistical differences disappeared. RT in the CSVD group was significantly thinner than that in the control group (OR (95% CI): 0.960 (0.923, 0.999), *p* = 0.043), and the statistical difference persisted after adjusting for gender, age, and medical histories (OR (95% CI): 0.954 (0.912, 0.997), *p* = 0.037). There were no significant differences in the remaining retinal structure and vessel density metrics between the two groups (Table 3).

**Table 3:** Comparison of different retinal structure and vessel density metrics between CSVD and control group.

	Missing (*n*)	group		Crude		Adjusted	
		CSVD group	Control group	OR (95% CI)	*p* value	OR (95% CI)	*p* value
SRCP	21	48.4 (47.1, 51.3)	51.3 (48.4, 53.1)	0.831 (0.704, 0.982)	0.030	0.843 (0.702, 1.011)	0.066
DRCP	21	52.0 (45.9, 56.8)	52.2 (49.7, 56.6)	0.953 (0.880, 1.031)	0.299	0.968 (0.885, 1.058)	0.472
FD-300	24	12.8 (10.5, 13.3)	13.0 (12.0, 13.8)	0.668 (0.460, 0.970)	0.034	0.767 (0.506, 1.165)	0.214
FAZ	24	0.3 ± 0.1	0.3 ± 0.1	1.930 (0.016, 236.745)	0.789	2.497 (0.007, 945.924)	0.763
RPC	12	49.2 ± 5.7	52.0 ± 5.0	0.910 (0.832, 0.996)	0.040	0.930 (0.843, 1.027)	0.152
RT	3	269.9 (259.8, 278.2)	274.4 (271.2, 280.2)	0.960 (0.923, 0.999)	0.043	0.954 (0.912, 0.997)	0.037
GCC	3	96.8 (91.6, 102.0)	98.4 (93.1, 102.0)	0.964 (0.911, 1.021)	0.208	0.964 (0.902, 1.030)	0.281
RNFL	9	102.2 (92.1, 108.3)	102.4 (95.8, 106.7)	0.980 (0.946, 1.015)	0.251	0.986 (0.943, 1.031)	0.526

Adjusted: adjusted by age, gender, medical history of cerebrovascular disease, hypertension, diabetes, hyperlipidemia, and cognitive impairment. SRCP, superficial retinal capillary plexus; DRCP, deep retinal capillary plexus; FAZ, foveal avascular zone; FD-300, foveal density- 300; RPC, radial peripapillary capillary; RT, retinal thickness; GCC, ganglion cell complex; RNFL, retinal nerve fiber layer.

**Figure 2:**
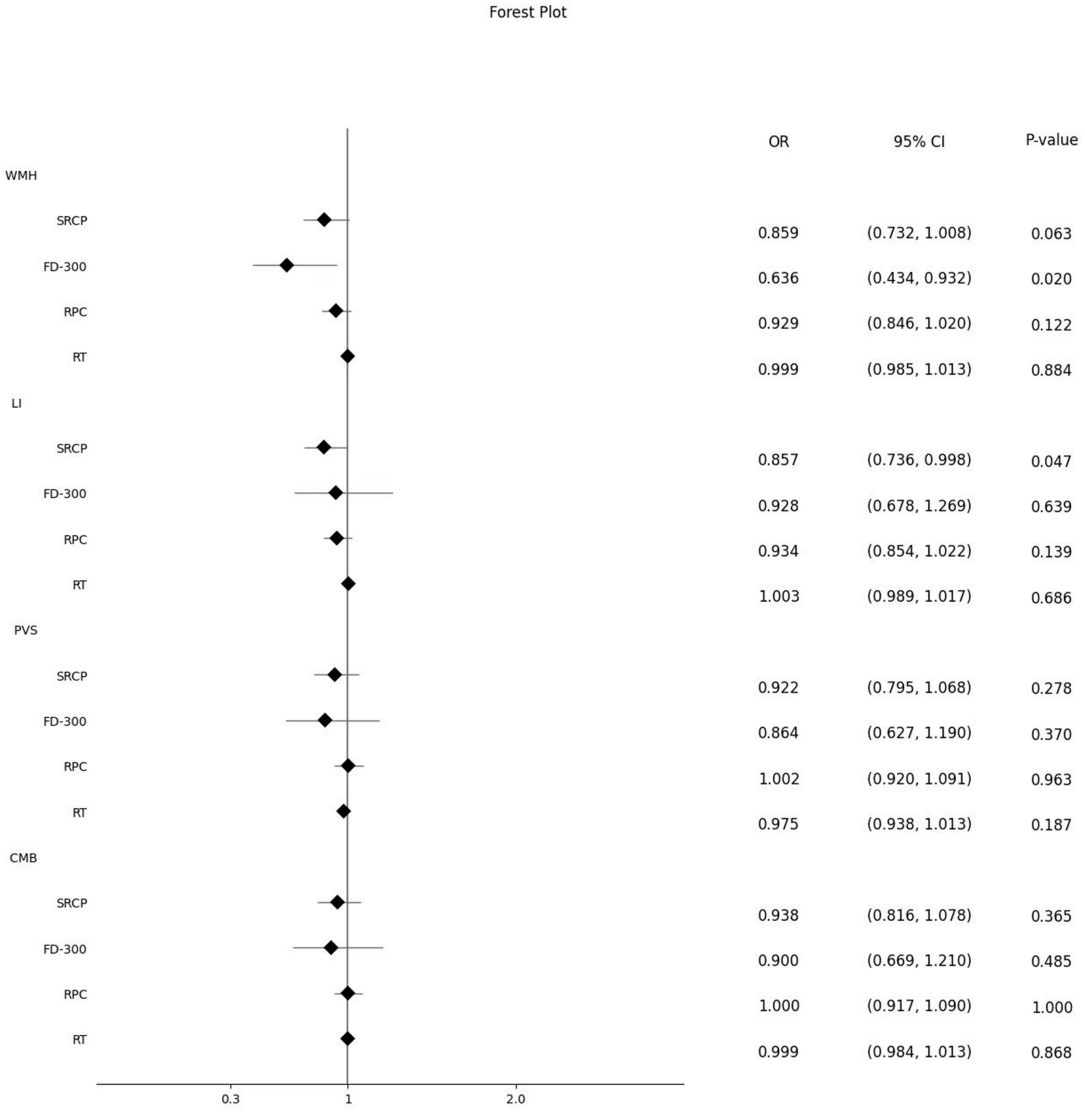
Effect of different ophthalmic metrics on different CSVD imaging manifestations. WMH, white matter hypertensions; SRCP, superficial retinal capillary plexus; FD-300, foveal density- 300 length; RPC, radial peripapillary capillary; RT, retinal thickness; LI, lacunar infarctions; PVS, perivascular spaces; CMB, cerebral microbleeds.

Next, we further analyzed the changes in ophthalmic metrics of the patients grouped according to different CSVD imaging manifestations and scores. It was found that after adjusting for related risk factors, SRCP vessel density showed a significant downward trend with the increase of CSVD scores (β: −0.087, 95%CI: −0.166, −0.008, *p* = 0.031) (Table 4), and this vessel density was also significantly lower in patients with LI than in those without LI [OR (95% CI): 0.857 (0.736, 0.998), *p* = 0.047]. FD-300 vessel density was significantly lower in patients with moderate-to-severe WMH than in those without [OR (95% CI): 0.636 (0.434, 0.932), *p* = 0.020]. There were no significant differences in the main ophthalmic metrics between patients with or without PVS and CMB (Figure 2).

**Table 4:** Comparison of different retinal structure and vessel density metrics stratified by CSVD scores.

	CSVD scores					β	95% CI	*p* value
	0	1	2	3	4			
*n*	29	26	15	21	9			
SRCP	51.3 (48.4,53.1)	50.7 (48.1,52.4)	47.7 (44.4,50.4)	48.1 (47.5,52.4)	47.0 (43.0,48.9)	−0.087	−0.166, −0.008	0.031
FD-300	13.0 (12.0,13.8)	13.0 (11.5,13.8)	11.8 (10.9,12.9)	12.8 (10.4,13.2)	9.9 (8.9,13.2)	−0.149	−0.323, 0.026	0.094
RPC	51.4 (49.3,56.2)	49.4 (44.6,55.5)	48.4 (44.1,53.2)	50.0 (43.9,54.3)	48.4 (44.6,50.5)	−0.026	−0.076, 0.024	0.307
RT	274.4 (271.2, 280.2)	271.7 (265.1,277.5)	263.7 (259.7,275.0)	277.8 (265.1,282.1)	254.7 (240.5,268.4)	−0.002	−0.011, 0.007	0.652

Adjusted: adjusted by age, gender, medical history of cerebrovascular disease, hypertension, diabetes, hyperlipidemia, and cognitive impairment. SRCP, superficial retinal capillary plexus; FD-300, foveal density- 300; RPC, radial peripapillary capillary; RT, retinal thickness.

## Discussion

This study systematically analyzed the relationship between different CSVD imaging manifestations and various retinal structure and vessel-related metrics, which indicates observing alterations in retinal metrics has become an optional direction in exploring CSVD. OCT and OCTA devices are portable, time-saving, economical, and simple to operate, with results that can be automatically interpreted, making them more suitable than cranial MRI for examinations or screenings of CSVD in large populations in communities in the future.

Since retinal microvascular and intracranial small vessel are relevant, some retinal microvascular diseases have also shared similar risk factors with CSVD and cerebrovascular diseases, such as age, hypertension, diabetes (Cannistraro et al., 2019; Chojdak-Lukasiewicz et al., 2021; Poli and Yusuf, 2021; Deng et al., 2022; Klyscz et al., 2023). OCTA is a prominent non-invasive and rapid method for detecting blood perfusion in retinal microvessels at different layers and locations. Studies on the relationship between different OCTA indicators and CSVD are limited and have suggested that some retinal vessel densities, such as SRCP and RPC, are related to certain imaging manifestations of CSVD (Lee et al., 2020; Wang et al., 2021; Ma et al., 2022). Compared to these studies, we analyzed various indicators related to retinal microvascular vessel density more systematically and found that SRCP vessel density is closely related to the severity of CSVD imaging manifestations. Retinal vessels are distributed differently in various parts of the fundus, and their blood flow autoregulation capabilities also vary. Young people have stronger retinal blood circulation autoregulation capabilities than older people. Less severe ischemia can be compensated for by autoregulation without causing symptoms (Chen et al., 2019). For different layers, SRCP has stronger autoregulation capabilities than DRCP. When retinal ischemia is mild and hemodynamics are unstable, functional compensation is first achieved through SRCP autoregulation, which explains why SRCP is more sensitive in reflecting retinal ischemia (Wang et al., 2021).

Our study also found differential expressions between retinal vessel density at different CSVD imaging manifestations, possibly due to their different pathophysiological mechanisms (Liew et al., 2014). Changes in SRCP vessel density are often related to small arteriole diseases. In our study, as the CSVD score of the patients increased, suggesting more severe intracranial small vessel lesions, the presence or absence of lacunar infarctions was closely related to changes in SRCP vessel density. This indicates that the occurrence and development of CSVD are similar to changes in retinal superficial layer vessel density and may be related to the pathophysiological changes of small arteriosclerosis, which is consistent with some published studies (Wang et al., 2021). Changes in retinal metrics are closely related to the neuroimaging changes of CSVD. The reason for this may be that these vascular and structural changes, similar to cranial MR imaging changes, are more reflective of the pathological changes and pathogenesis of CSVD, rather than as an assessment tool for the clinical manifestations of CSVD. In addition, previous studies have reported a correlation between FAZ and its surrounding vessel density with CSVD (Xu et al., 2023). We also found a close correlation between FD-300 and moderate- severe WMH, which to some extent indicates the relationship between retinal ischemia and WMH.

In previous studies, we found that changes in RNFL thickness were closely related to stroke events, and some studies have also suggested the relationship between RNFL and GCC thickness changes and different imaging changes in CSVD (Wang et al., 2014; Simsek, 2017; Langner et al., 2022). Since SRCP is mainly distributed in the RNFL and GCC layers, there should theoretically be consistency between the two changes. However, in our study, we did not find statistically significant differences in RNFL thickness changes and GCC thickness changes between the two groups of patients with and without CSVD imaging manifestations. We only found that the average retinal thickness was significantly thinner in the CSVD group. In future studies, we plan to analyze the changes in retinal structure thickness in different quadrant regions more carefully to discover more meaningful results. Furthermore, previous researches have confirmed that alterations in the vascular and structural components of the retina may also be related to other diseases of the nervous system, such as Parkinson’s disease (Robbins et al., 2021), amyotrophic lateral sclerosis (Cennamo et al., 2022), and neuromyelitis optica (Oertel et al., 2021), etc. Analyzing the reason for this, it is considered that the retina and the central nervous system have the same embryonic origin, the retina may be an extension of the central nervous system. Therefore, its pathological changes could mirror abnormalities in the brain’s nervous system.

This study has some limitations. Firstly, the study has a limited enrolled population, resulting in a certain bias in the gender, group allocation, and the proportion of the CSVD scores of the enrolled individuals. In addition, the study population consisted of inpatients in the vascular neurology department of our hospital. Since most inpatients already have related disease symptoms, we could not observe changes in retinal vessel density and other indicators in subclinical or asymptomatic CSVD patients, resulting in selection bias. Secondly, in the analysis of the results of the enrolled patients, we did not obtain more detailed research results, such as the comparison of the relationship between different quadrant retinal vessel density, retinal layer thickness changes, and the development of CSVD. In future studies, we plan to further divide the various indicators into different quadrant regions for more detailed analysis. Thirdly, this study focused on the relationship between different imaging manifestations of CSVD and various ophthalmic indicators, without strictly recording the clinical manifestations related to CSVD in the enrolled patients, such as cognitive impairment, stroke symptoms, and gait abnormalities. There is a lack of comprehensive analysis and interpretation. Therefore, we look forward to further in-depth, comprehensive, and systematic understanding of the relationship between CSVD and the retina in future research, providing new ideas and directions for the occurrence and development of CSVD.

## Conclusion

Different retinal structure and vessel densities may associate with the occurrence and severity of total CSVD scores and its different radiological manifestations. Exploring CSVD by observing alterations in retinal metrics has become an optional research direction in future.

## Data availability statement

The raw data supporting the conclusions of this article will be made available by the authors, without undue reservation.

## Ethics statement

The studies involving humans were approved by Beijing Tiantan Hospital, Capital Medical University, Beijing, China. The studies were conducted in accordance with the local legislation and institutional requirements. The participants provided their written informed consent to participate in this study.

## Author contributions

DW: Conceptualization, Funding acquisition, Writing – original draft. LW: Conceptualization, Methodology, Writing – original draft. JW: Investigation, Methodology, Project administration, Writing – review & editing. YD: Formal analysis, Software, Writing – review & editing. KW: Data curation, Visualization, Writing – review & editing. MW: Investigation, Methodology, Writing – review & editing. LY: Resources, Supervision, Writing – review & editing. XZ: Conceptualization, Project administration, Supervision, Validation, Visualization, Writing – review & editing.
